# Atmospheric Deposition of Phosphorus to the Everglades: Concepts, Constraints, and Published Deposition Rates for Ecosystem Management

**DOI:** 10.1100/tsw.2002.813

**Published:** 2002-07-03

**Authors:** Garth W. Redfield

**Affiliations:** Environmental Monitoring and Assessment Department, South Florida Water Management District, 3301 Gun Club Road, West Palm Beach, FL 33406, USA

**Keywords:** atmospheric phosphorus deposition, phosphorus inputs, bulk precipitation, atmospheric particles, conceptual deposition model, dry deposition, nutrient loading and giant atmospheric particles

## Abstract

This paper summarizes concepts underlying the atmospheric input of phosphorus (P) to ecosystems, published rates of P deposition, measurement methods, and approaches to future monitoring and research. P conveyed through the atmosphere can be a significant nutrient source for some freshwater and marine ecosystems. Particle sources and sinks at the land-air interface produce variation in P deposition from the atmosphere across temporal and spatial scales. Natural plant canopies can affect deposition rates by changing the physical environment and surface area for particle deposition. Land-use patterns can alter P deposition rates by changing particle concentrations in the atmosphere. The vast majority of P in dry atmospheric deposition is conveyed by coarse (2.5 to 10 μm) and giant (10 to 100 μm) particles, and yet these size fractions represent a challenge for long-term atmospheric monitoring in the absence of accepted methods for routine sampling. Most information on P deposition is from bulk precipitation collectors and wet/dry bucket sampling, both with questionable precision and accuracy. Most published annual rates of P deposition are gross estimates derived from bulk precipitation sampling in locations around the globe and range from about 5 to well over 100 mg P m year, although most inland ecosystems receive between 20 and 80 mg P m year. Rates below 30 mg P m year are found in remote areas and near coastlines. Intermediate rates of 30 to 50 mg P m year are associated with forests or mixed land use, and rates of 50 to 100 mg P m year or more are often recorded from urban or agricultural settings. Comparison with other methods suggests that these bulk precipitation estimates provide crude boundaries around actual P deposition rates for various land uses. However, data screening cannot remove all positive bias caused by contamination of bucket or bulk collectors. As a consequence, continued sampling with these standard collectors in a region will not reduce the large uncertainty in rates derived from existing data. Calibrated surface accumulation methods hold promise as a primary means to estimate P flux in future monitoring. New methods for long-term P deposition monitoring will require an intercomparison of P flux estimates from surrogate surfaces, impactor sampling of particle concentrations combined with deposition models, and “throughfall” estimates for natural canopies. With better sampling methods and more long-term monitoring data, the importance of atmospheric P deposition in ecosystem dynamics and management can be better understood and predicted.

## INTRODUCTION

The flux, or loading, of nutrients to ecosystems occurs primarily through point source discharges, surface water, groundwater, and atmospheric deposition. The input of nutrients from wastewater discharges has become less of a problem over the last 25 years and can be quantified from discharge monitoring records. Loading by surface water runoff has been measured for many ecosystems and can be the major source of nutrients for aquatic environments, particularly those with large watersheds. Groundwater inputs are more difficult to assess and are often estimated indirectly by mass balance calculations. Nutrients conveyed by rainfall and dry fallout from the atmosphere can be a substantial source of nutrient loading to some freshwater environments[1,2,3,4,5,6]. However, atmospheric inputs will only be of quantitative importance for freshwater ecosystems that are oligotrophic with low rates of nutrient loading from internal and external sources. For the marine environment, Graham and Duce[7], Jickells[8], and Guerzoni[9] have emphasized the overall importance of atmospheric inputs to the oceans, particularly in oligotrophic regions. Conversely, the oceans may contribute substantial amounts of P to continents through aerosols injected into the air from the ocean surface[10].

Compared to other elements conveyed through the atmosphere, P has received relatively little attention. Surprisingly, Gorham's[1] review of the chemistry of inland waters does not include P in the discussion of the chemistry of atmospheric precipitation. The multiagency Great Waters Program[11], Environmental Protection Agency (EPA), and National Oceanic and Atmospheric Association (NOAA) dry deposition networks[12], and the National Atmospheric Deposition Program/National Trends Network[13] do not include total P monitoring from atmospheric sources. The U.S. Environmental Protection Agency[14], in a thorough review of atmospheric particles, included information on P deposition in only a few tables concerning particle composition. In addition, estimates of atmospheric P loading with methods other than bulk and wet/dry bucket collectors are not common because many studies of atmospheric inputs have been focused on aerosols associated with acidic deposition, such as nitrogen and sulfur[12,15].

P is limiting to algal growth in many freshwater lakes[16,17], and P control is often the primary focus of lake management[18,19]. In large, shallow, freshwater environments, such as the Florida Everglades, atmospheric inputs of P could be very important because a major portion of this expansive marsh ecosystem receives very little water and nutrient input from surface water inflow[20].

Substantial uncertainty exists in both the precision and accuracy of deposition rates for P in South Florida and elsewhere. Using an extensive 13-station data set from South Florida, Ahn and James[21] demonstrated high variability in loading estimates for atmospheric P and concluded that this substantial uncertainty was due to random noise in the data. Irwin and Kirkland[22] reported a wide range of values in their review of the historical data on precipitation quality (bulk deposition) in Florida and concluded that these data were insufficient to define baseline conditions for the region. Hendry et al.[23] also reported major variation in bulk P deposition among sampling sites, with values ranging from 17 to 111 mg P $m^{-2}$ $year^{-1}$. They found the statewide average bulk deposition rate to be 50 mg P $m^{-2}$ $year^{-1}$ and reported wet/dry collector rates from 24 to 96 mg P $m^{-2}$ $year^{-1}$. More recently, Dixon et al.[24] reported annual station averages ranging from 52 to 176 mg P $m^{-2}$ $year^{-1}$ for bulk deposition in the Tampa Bay area, with a seven-station average of 93 mg P $m^{-2}$ $year^{-1}$. Peters and Reese[25] also found data variability to be very high (wet values from 0.001 to 0.620 mg P $l^{-1}$) in dry and bulk bucket-type collectors near Lake Okeechobee in Florida. Such variability is not unique to Florida; published deposition rates for P in other regions[26,27,28,29] range from less than 10 to well over 150 mg P $m^{-2}$ $year^{-1}$. An extensive compilation of such rates is included in this review.

While high variability is common for environmental parameters, it is compounded for P deposition by the lack of information on the accuracy of data used in estimating long-term ecosystem inputs. Standard methods of atmospheric monitoring, particularly those for particle fallout, often measure only a portion of total deposition. Some approaches provide defensible estimates for the deposition of fine aerosols (micrometeorological methods), but do not quantify the fallout of larger particles[30]. The common approach to long-term flux measurement using funnels or bucket collectors provides the opposite result. Bucket devices provide some data on the flux of larger particles, but are subject to great uncertainty due to their poor aerodynamic qualities and susceptibility to environmental contamination. Methods for measuring compliance with air quality standards assess ambient concentrations of fine particles that affect human health, but are not suitable for deriving total fallout to the environment[31]. Those studies that have provided rigorous estimates of particle flux used several complementary collection devices simultaneously, but were limited to short-term estimates in only a few environments[32,33]. The limitations of methods and constraints on study design, as well as other factors to be discussed later in this paper, require the ecosystem scientist or manager to interpret disparate published rates of atmospheric material flux from a large body of literature, with substantial variability and minimal assurance of accuracy.

There are many natural sources of variability in atmospheric deposition[34]. For wet deposition, rainfall volume, season, and storm path can alter mass deposited in an event[35]. Dry deposition can be affected by the size spectrum of depositing particles, properties of collecting surfaces, and season[36], as well as wind speed[32,37]. Another key factor is sample contamination from nonatmospheric natural debris or that associated with birds or insects attracted to the collector device[25,38,39,40,41,42]. As a result of these factors, atmospheric turbulence and boundary layer phenomena, dry rates are expected to be more variable than those for wet deposition[12,34,43].

The lack of precision and accuracy of dust deposition data in general and of P data in particular presents an immediate challenge for environmental management. For example[44], the vast majority of the remnant Florida Everglades is very shallow and often displays ambient P levels at or below 10 µg $l^{-1}$. Atmospheric loading in the middle or upper range of published values could be a significant source of enrichment to portions of this ecosystem[42,45]. The Everglades Forever Act, passed by the Florida Legislature in 1994, requires the South Florida Water Management District, in cooperation with several other state and federal agencies, to restore the remaining Everglades ecosystem. The control of P loading to this oligotrophic, subtropical environment is a cornerstone of the restoration. However, P-loading estimates from the atmosphere for any location or for the region as a whole have a high degree of uncertainty. This lack of definitive information on P inputs from the atmosphere provides the fundamental motivation for this study. Gordon[46] stressed the need for atmospheric science to provide improved estimates of nutrient deposition to ecosystems. Gardner[47] recommended more attention to P sources, mechanisms of entrainment and modes of atmospheric deposition to better assess the importance of atmospheric P. Finally, Lovett[34] called for more attention to atmospheric deposition measurements and emphasized the need to reconcile methodological uncertainties.

This review attempts to bridge the environmental and atmospheric sciences to convey information on the atmospheric deposition of P. The first objective of this paper is to provide a conceptual framework for information on P deposition in rainfall and dustfall based on published data. The second objective is to summarize P deposition rates in this conceptual framework and provide reasonable bounds for P deposition rates to aquatic ecosystems. Interpretation of this information is bolstered by a review of background deposition rates to remote areas of the globe. Estimates of P deposition using nontraditional methods are also considered as a source of independent evidence on the accuracy of published values for application to environmental management and to South Florida, specifically. The third objective is to summarize available information on field methods that might be used to improve estimates of atmospheric loading of P to aquatic ecosystems. Finally, this paper concludes with a consideration of the circumstances, under which P deposition monitoring may reduce uncertainty for environmental decision making, considering constraints inherent in its quantification. The information analyzed to meet these objectives should be useful to environmental managers and ecosystem scientists worldwide as they seek to understand the sources and sinks of P in systems where nutrient enrichment is a target of environmental management. The environment of South Florida is used as a case study for illustrating concepts of atmospheric deposition and suspension. Vast areas of the Everglades receive little water directly from surface inflows and may have been influenced over many years by atmospheric deposition.

## MAJOR FEATURES OF THE SOUTH FLORIDA ENVIRONMENT

Because examples from South Florida are used frequently in this review, the reader will benefit from a general understanding of the regional ecosystem and its geography. The once vast Everglades landscape is now a highly modified wetland system known collectively as the Everglades Protection Area for the purposes of the restorative Everglades Forever Act of 1994 (§373.4592, Florida Statutes). The remnant Everglades is composed of four areas (Fig. 1), and atmospheric deposition is expected to be important to the long-term nutrient status of interior marshes. Water Conservation Area 1 (WCA-1), the Arthur R. Marshall Loxahatchee National Wildlife Refuge (Refuge), consists of 566 km$^2$ of wetlands. This area receives runoff from the neighboring Everglades Agricultural Area to the northwest. Water from WCA-1 flows southward into Water Conservation Area 2 (WCA-2). This area of 538 km$^2$ also receives surface water inflow from the Everglades Agricultural Area. WCA-2 has been the subject of intensive eutrophication research and water quality monitoring in the last three decades[48].

Water Conservation Area 3 (WCA-3; Fig. 1), the largest of the WCAs at 2,342 km$^2$, contains tree islands, wet prairies, and sloughs scattered across a vast sawgrass marsh. Water from WCA-3 flows southward into Everglades National Park with its 5,569 km$^2$ of highly valued aquatic sloughs, marl-forming marshes, and mangroves. Vast areas of the park have very low total phosphorus levels in surface water (<10 μg l$^{-1}$) and receive most water from local rainfall.

## CONCEPTUAL BASIS FOR P DEPOSITION FROM THE ATMOSPHERE

### Components and Size Fractions

Atmospheric deposition of substances like P occurs as wet, dry, and cloud deposition[34]. Wet deposition is associated with directly falling atmospheric precipitation containing gases or particles and varies with individual rainfall events, spatial patterns of precipitation, and processes involved

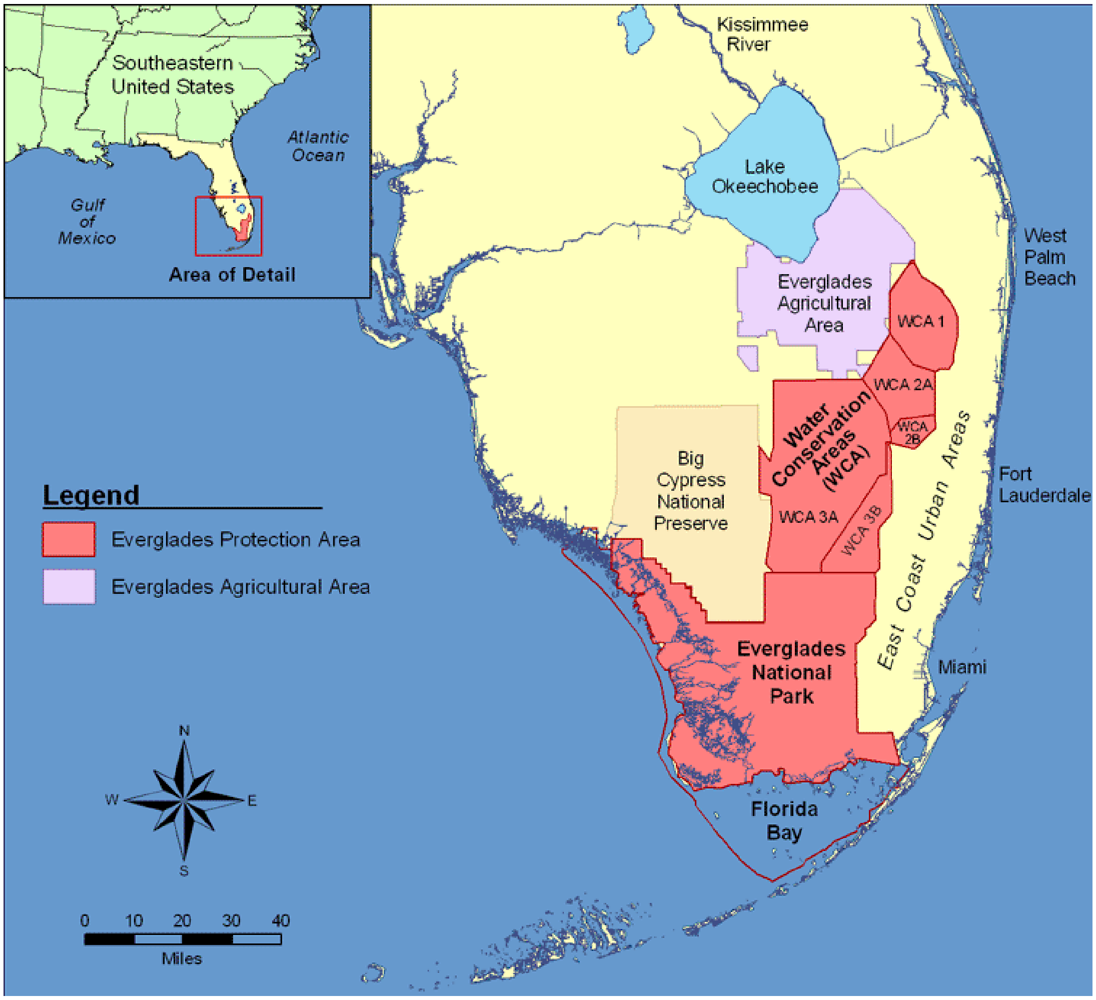

**FIGURE 1.** Major geographic features of South Florida. The Everglades Protection Area encompasses the remnant Florida Everglades and is compromised of three Water Conservation Areas and Everglades National Park. Water resources in the region are the responsibility of the South Florida Water Management District, West Palm Beach, FL.

in scavenging of materials from the air. The separation between dry and wet deposition is defined operationally by the common Aerochem Metrics®, Inc. wet/dry collection system that opens the wet bucket only when rain is falling in substantial amounts. Hicks et al.[12] define dry deposition as "the gravitational settling of large particles not associated with falling precipitation, and the turbulent exchange of small particles and trace gases to the surface." Dry deposition rates are determined by wholly different mechanisms from wet deposition, including particle size and concentration, settling velocity and nature of the collecting surface. Dry deposition is more complex and variable than wet[43], and is very important in conveying particles to the land surface[34].

Cloud deposition occurs when fog or clouds contact surfaces and is important in elevated coastal or mountainous environments. Deposition from clouds and fog may be difficult to separate into wet and dry components; on a practical basis, however, it is measured by dry techniques. Cloud deposition will not be considered further in this analysis as a distinct process because sampling the dry and wet components assumes that materials conveyed by clouds and fog are captured. Also, dew will be considered as a factor altering dry deposition through surface wetting, as suggested by Lovett[34], rather than as a separate form of deposition.

P is conveyed through the atmosphere almost entirely by suspended particles. However, there is a gaseous component to the P cycle involving phosphine ($PH_3$), diphosphine ($P_2H_4$), and phosphorus pentoxide ($P_2O_5$) that may be an important vehicle for atmospheric P transport under some circumstances. $PH_3$ gas can be emitted from anaerobic environments in which energy and phosphate are available, such as sewage plants, marsh and river sediments, landfills, or composting soils[49]. Devai et al.[50] found that up to 50% of the P mass balance deficit for stabilization lakes associated with two sewage treatments plants could be explained by the emission of $PH_3$ gas into the atmosphere. Measurements in marsh sediments, including samples from the Everglades, confirmed that $PH_3$ can be emitted from natural marsh sediments under strong reducing conditions, and emission rates can be enhanced when given additional P and an energy source[51]. Newman[27] mentions another potential gaseous pathway when plant P is oxidized to $P_2O_5$ and vaporized during biomass burning. All these compounds will form phosphoric acid when they contact water in the air and will deliver P to an ecosystem as phosphate, a biologically available form. While existing evidence suggests that the gaseous phase of the P cycle is ubiquitous, the quantitative importance of $PH_3$, $P_2H_4$, and $P_2O_5$ to atmospheric deposition is not known. Gaseous forms of P are worthy of additional attention, particularly in the vicinity of landfills, eutrophic marshes, and sewage treatment plants.

The dry fallout of P is due almost entirely to particles greater than 2 μm in diameter[7,52]. P can be conveyed in large particles derived from soils or biota, including pollen, spores, waxes, resins, and microbes[53,54]. More recently, several studies have demonstrated clearly that dry deposition flux of crustal elements is almost entirely derived from large atmospheric particles[32,33,55,56,57]. Although the following discussion is organized around the three common size fractions of atmospheric particles (fine, coarse, and giant; Table 1), a conceptual framework for P dry deposition must concentrate on the nature and behavior of larger coarse and giant atmospheric particles. General information on atmospheric particles can be found in several recent sources[31,54,58,59].

Fine particles (<2.5 μm in diameter) (Table 1) may have residence times in the atmosphere ranging from weeks to months, depending on mixing and removal processes[60]. The formation of secondary particles from smaller ones decreases the relative proportion of the smallest atmospheric particles[58,59]. Simultaneously, coarse and giant particles are lost from the atmosphere at higher rates. The deposition of aerosols thousands of kilometers from sources reflects the net effect of multiple processes. Studies of such transport suggest that factors other than gravitational settling maintain particle suspension in the atmosphere and allow long distance movement of particles[61,62].

Giant particles (10 to 100 μm, Table 1) have high settling rates based on the balance between gravitational settling and viscous atmospheric drag predicted by Stokes' Law; Noll and Fang[63], Lin et al.[56], and Caffrey et al.[57] provide a guide to information on settling rates. It is tempting to conclude that such high loss rates would preclude giant particles from traveling far from sources. However, evidence suggests that atmospheric processes can result in longer suspension and transport than physical principles would suggest. Betzer et al.[62] reported substantial mass flux of giant (> 75 μm) atmospheric particles at remote marine locations in the North Pacific Ocean. Betzer et al.[62] could not explain how such particles could travel over 10,000 km from China, but they suspect that resuspension by convective storms may be involved. Ellis and Merrill[61] also found Stokes' Law predictions did not accurately predict long-range transport to Barbados during certain times of the year. Through analyses using a model to predict dry deposition rates, Noll and Fang[63] concluded that larger (ca. 15 μm) particles tend to respond more to increased wind speeds than smaller particles and therefore have the potential to remain in the atmosphere longer than might be predicted by Stokes' Law of settling velocities. There is other evidence from *in situ* measurements that larger particles stay aloft long enough to dominate dry deposition far from sources. For example, Holsen et al.[55] found that over 98% of dry deposition to Lake Michigan was due to particles greater than 6.5 μm. Analysis of data from Chen et al.[64] reveals that over 85% of P was conveyed by either coarse or giant particles in maritime breezes.

Davidson et al.[65,66], Noll and Fang[63], Lin et al.[56], and Zufall et al.[33] provide overwhelming evidence that the vast majority of dry deposition mass flux is conveyed by giant particles

**TABLE 1**
**The Characteristics of Particle Size Categories Conveying Atmospheric P**

| **Characteristic** | **Particle Size Category*** | | |
|---|---|---|---|
| **Key Attributes for P Deposition** | **Fine Particles (<2.5 µm)**** | **Coarse Particles (2.5–10 µm)**** | **Giant Particles (10–100+ µm)** |
| Scale of transport | Often global/regional; long atmospheric suspension possible | Often regional; intercontinental transport of dusts well documented | Often regional/local; global episodically; mechanisms of suspension unclear |
| Common sources | Combustion, air transformations, organic compounds, microbes | Fugitive dusts, biotic processes, sea spray, volcanic ash | Soil, coal, vehicular, and industrial dusts, organic debris, sea spray |
| Land-use association | Low density; low landscape disturbance | Urban, industrial, or agriculture | Dependent on local groundcover and biological communities |
| P content | High, emission sources vary (0.1–1.0 % P) | Crustal levels likely (0.1–0.2 % P) | Variable based on particle source |
| Transport and deposition mechanisms | Follow local air eddies; Brownian diffusion and surface adhesion can be important | Less dependent on eddies; interception and inertial effects important | Gravitational and density effects dominate settling and surface adhesion |
| Deposition velocity | Low, ca. 0.01–0.1cm $s^{-1}$ | Moderate (ca. 0.1–1.0 cm $s^{-1}$) | High (ca.1 – >10 cm $s^{-1}$); density and shape effects possible |
| Surface effects | Surface fine structure and wetness are important | Variable effects, wetness important, inertia involved | Inertial and gravitational forces dominate |
| Canopy effects | Important as sink; biochemical release uncertain source; leaf area alters deposition | Variable; resuspension, creation and bounce-off possible | Important as source, esp. biotic particles; canopy structure less important |
| General linkage to P deposition rate | Overall, minor contributor to P flux; may support back-ground deposition | Important component of total P flux; diel, seasonal and climatic cycles | Dominates P flux; diel, seasonal and episodic variation; desert storms |

* Size of groups of particles is usually expressed as aerodynamic diameter; the diameter of a sphere with a settling velocity at a density of 1 g $cm^{-3}$ equal to that being considered.

** The fine and coarse size categories follow those developed for particulate matter monitoring and assessment by the U.S. Environmental Protection Agency[14]. The giant particle category is not relevant for air pollution assessment based on human health considerations and cannot be measured by most high-volume compliance samplers that have a cutoff point of about 10 µm.

The information in this table was drawn from various sources in the literature, particularly Finlayson-Pitts and Pitts[58], Noll and Fang[63], Hicks et al.[12], Lovett[34], Chow[31] and the U.S. Environmental Protection Agency[54].

between about 10 and 100 µm. In addition, Brook et al.[67] found that particles greater than 10 µm represented about 44% of total suspended particles for 14 urban monitoring stations across Canada, while Lundgren et al.[68] noted substantial particle mass occurring in sizes greater than about

15 μm in 5 U.S. cities. Paode et al.[69] provide evidence that the dry deposition of lead, copper, and zinc, traditionally thought to be conveyed by fine particles, is dominated by particle flux from coarse and giant particles. Although giant particles are usually present in low concentrations, their higher sedimentation rates and high mass per particle provide for high deposition flux. These findings on the importance of giant dust particles to dry deposition rates should apply equally to P-bearing particles.

Defining which particle size is truly atmospheric is not a straightforward process. The dilemma, as emphasized by Hicks et al.[12], is how to decide what particulate matter is a natural part of atmospheric deposition and what is caused by contamination of the sampler or by local, nonatmospheric inputs. The objective of measuring P deposition for environmental management or research is to estimate total input of P to an ecosystem. Any pragmatic size cutoff point for atmospheric particles must consider inputs that could be missed as a result of the criterion. Studies dating from the 1950s suggest that natural organic matter from the air may be a very important source of carbon or other elements[1,70]. Ecological studies have reported that particulate debris up to 1 μm in diameter can represent an important source of depositional P[5,71,72]. The importance of debris inputs may be scale dependent and small aquatic ecosystems surrounded by forest may be exceptional in the amount of material delivered via air transport of large particulate detritus. In fact, there are estimates of substantial deposition from airborne particles greater than 1 mm in diameter, known as *large particulate organic matter* in the ecological literature[73,74].

Natural detritus or very large dust particles in the air are difficult to separate from local recycling and sample contamination by bird excrement, insects, or organic matter. For most systems, atmospheric transport should be considered an important mechanism for particles of 100 μm or less in diameter. Some bulk deposition sampling programs preclude all larger materials from samples by physical means: van Ek and Draaijers[43] and Anttila et al.[75] used filtration to remove debris from samples; Brown and Lund[76] used cotton in funnels to keep larger materials out of samples. Other sampling programs use netting to avoid all particles greater than about 1 mm[2,77]. Most particles above 100 μm in size will travel relatively short distances and for most larger ecosystems should not represent a significant component of deposition. As a result, using collection methods that do not sample particles greater than 100 μm in size should not interfere with estimates of atmospheric fallout.

## Major Sources of Variation in P Deposition Rates

Many processes lead to natural changes in atmospheric particle concentrations and deposition rates (Table 1). Change in particle sources is an obvious cause of variation in atmospheric P inputs to ecosystems. P-bearing particles are derived largely from manufacturing, soil, pollen, insects, and other biological debris, fertilizers, volcanic ashes, combustion of coal and other fossil fuels, organophosphate pesticides, and aerosols from the ocean[26,27]. P can be conveyed through exhaust from vehicles or incinerators when many common materials are burned, including some complex organic compounds. Many sources of atmospheric P are altered by human activities, and it is, therefore, not surprising that long-term variations in dust concentrations in the atmosphere can be traced to land-use changes; Prospero et al.[79] even consider emitted dust as a global pollutant.

Unfortunately, unnatural variation in rates from local contamination is a very common problem and can dominate values generated by long-term sampling with surrogate surface collectors, particularly in productive wetland environments. Screening or sample filtration cannot prevent occasional contamination, and censoring of samples that have been compromised by contamination is essential to estimating deposition rates. Using data from wet/dry bucket monitoring in South Florida, Ahn[38,39] and Ahn and James[21,40,41] provide quantitative means for detecting outlying data, filling data gaps through statistical modeling and assessing uncertainty in data compromised by contamination. The mean deposition rate these authors developed was quite reasonable, but the general lack of temporal or spatial signals in the postcensored data suggests that the statistical removal of contaminated data was not able to clarify the underlying deposition signal.

Walker[42] estimated P deposition to the Arthur R. Marshall Loxahatchee National Wildlife Refuge in the northern Everglades (Fig. 1) by assessing the sensitivity of both wet and dry data to various sources of contamination. He conducted a thorough analysis of effects of contamination on deposition estimates from each of the four marsh stations and found that droppings, vegetation, insect debris, and spider webs were common sources of contamination. Even with many contaminated samples removed, rates from weekly data still displayed variation over about two orders of magnitude. The statistical analyses of both Walker[42] and Ahn and James[21] document that *post hoc* statistical treatment removes some variation generated by extraneous material, but is not able to effectively remove noise and leave a contaminant-free signal by censoring. As a result, any long-term data from bulk, wet/dry, or other surrogate surface devices, including those reviewed in this study, yield data with some unrepresentative high values and rate estimates that are likely to be biased high.

Another important source of variation in P deposition flux is the elemental composition of depositing particles. The P content of atmospheric dust particles ranges from about 0.02 to over 2.0% of the mass, and varies with particle source. An extensive review by Chow[31] revealed that P in particles from road dust, soil, vehicles, and power plants occurs at levels between 0.1 and 1.0%. Particles of crustal origin can expected to contain P at about 0.1 to 0.2%[26]; Prospero et al.[79] found 0.105% to be a defensible average for estimating P transport from data on dust. Avila et al.[80] reported the P content of red rain dusts from the soils of North Africa to be 0.063%. Fine particles in exhaust from gasoline-powered cars were found to contain 0.11% P, and diesel exhaust contained 0.06% P[81]. Olmez et al.[82] provided data showing high P levels in particles from industrial sources. For the fine fraction, they found concentrations of 0.45 to 1.5%, and for coarse particles concentrations of 0.22 to 2.3%; particles from coal-fired power plants contained P at significant concentrations of 1.0 to 2.3%. Other biological particles have higher P content than crustal materials; 13 types of pollen showed an average of 1.6% P in a study by Banks and Nighswander[6]. For purposes of this study, a highly conservative value of 0.1% will be used to estimate P deposition from data on total particle mass.

Water surface effects can be very important to the deposition rates measured by various methods and can generate variation at the landscape scale. Both Cole et al.[72] and Jassby et al.[5] found that the wetted surface captured much more P than the same containers without water. Caffrey et al.[57], working on Lake Michigan, suggest that the lake surface can be expected to have higher rates of dry deposition than a flat surface due to surface roughness, whitecap formation, and humidity gradients. Zufall et al.[83,84] used modeling and wind tunnel measurements to confirm that wavy surfaces generally collect more particles than flat surfaces, and that giant particles may be less affected than smaller particles by the presence of waves. For elements such as P being conveyed in coarse and giant particles, water surfaces provide for less resuspension and bounce-off, resulting in greater net flux than a dry, flat surface. The net effect of all these processes remains uncertain, and more definitive information on deposition to water surfaces is needed to predict deposition rates for very large water surfaces, such as Florida's Lake Okeechobee and the Everglades.

Davidson et al.[37] stressed the importance of the dry collecting surface to deposition rates, and noted earlier work suggesting that deposition rates can vary over several orders of magnitude based on fine structure or geometry of the collecting surface. Thus, it would not be surprising to find significant differences in dry deposition rates in the same area within different plant canopies. The canopies tend to act as a sink for coarse and giant dust particles from the atmosphere[85], but may be a source of particles through resuspension and biological processes.

Edge effects occur both within and outside vegetation canopies. In forest stands deposition rates within five stand-heights of the edge are higher than those in the interior of the stand (reviewed by Erisman et al.[86]). Weathers et al.[87] reported that cloud deposition was reduced by 50% at

28 m from forest edges. Conversely, as one moves away from a canopy source, the deposition of plant-generated materials can be expected to decline greatly with distance from the stand edge[6,70,72,74,88]. This same phenomenon can be seen at a large scale in atmospheric P concentrations moving away from the coast; levels near Bermuda about 1,000 km offshore are approximately an order of magnitude less than P concentrations at the North American coastline[7]. For Southern Lake Huron, Delumyea and Petel[89] found across-lake gradients in P-bearing particles, and used these gradients in concentration to calculate deposition velocities for P. The challenge is how to estimate net deposition to natural canopies, such as the Florida Everglades. The Leaf Area Index has been found to be a quantitative means of estimating deposition to the forest floor[90] and clearly demonstrates that the capture of particles by plant canopies is important for any landscape-level estimate of deposition.

Processes responsible for particle concentrations in the atmosphere result in daily and seasonal variations in deposition rates. Deposition flux was found to be lower at night due to decreased wind velocity, lower particle concentrations, and a shift toward smaller particles[32,91,92]. Many researchers have reported that P deposition rates tend to be higher during the spring and summer[35,92,93,94]. Kopacek et al.[95] and Yang et al.[96] found deposition rates for P to be highest during spring and early summer. Finally, Prospero et al.[79] analyzed long-term data on dust concentrations in Barbados and found higher levels in summer and long-term changes associated with climatic cycles in North Africa. They concluded that dust transport varies with geographic sources and meteorological conditions, and produces clear seasonal and annual signals in particle concentrations spanning 27 years of sampling on the island of Barbados.

When all these factors are combined (Table 1), coarse and giant particle fluxes are expected to be quite variable and event related. Wind conditions are a major driving factor. Holsen et al.[55] concluded that Lake Michigan received most of its dry deposition during periods of strong southerly winds from the urban areas to the south of the lake. Lin et al.[56] provided direct evidence of high deposition rates associated with strong winds. The P deposition data of Bergametti et al.[97] also demonstrated episodic and seasonal signals for the northwestern Mediterranean Sea. Large particulate organic matter will generate variability in deposition rates stemming from transport during windy, high-energy events and/or pulses of biological production, such as pollen release, insect emergence, leaf abscission, epidemics of plant disease, or other natural occurrences. Wind speed and direction and other meteorological data should reduce noise to deposition data and help to account for variation in dry deposition rates.

## P Deposition: Integration from an Ecological Perspective

A conceptual model for South Florida provides some insight into the sources, sinks, and processes across the landscape (Fig. 2) and shows the dynamic nature of atmospheric particle concentrations and deposition rates spanning multiple temporal and spatial scales. Intercontinental and regional inputs are augmented by local sources, particularly those associated with urban or agricultural land uses, as air masses travel across the landscape. As this particulate matter is carried over a large natural area or water surface, such as the Everglades, larger particles are deposited at high settling velocities. Particle concentrations gradually decline as deposition exceeds suspension. Some particles will be emitted from natural sources, and resuspension of particles is possible. Several studies have suggested that materials can spiral across terrestrial systems, with the atmosphere as the agent of transport[66,98]. To the extent that this phenomenon occurs in atmospheric transport, it may involve active resuspension or simply the rebounding of larger particles[37]. Residence time in the atmosphere links time and space scales for sources and sinks of particles.

Considering the number of factors involved in particle production, transport, and sedimentation (Table 1; Fig. 2), temporal and spatial variability in P deposition rates are guaranteed. The timing of frontal or convective rainfall events will dictate when wet deposition occurs. Similarly for dry deposition, weather patterns and long-term climatic cycles will alter the trajectory of atmospheric

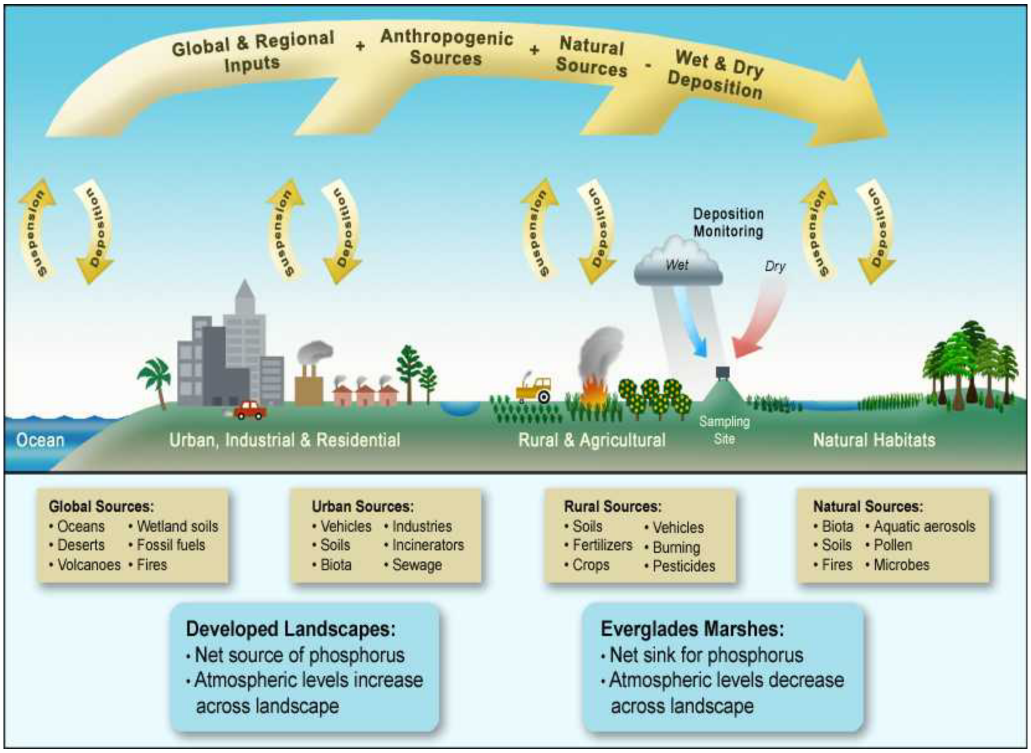

**FIGURE 2.** A conceptual model of the transport and deposition of phosphorus from the atmosphere. The landscape of South Florida (Fig. 1) is used for illustration, although sources, processes, and concepts can be applied to any landscape.

dusts from particle sources spread unevenly around the globe. The monitoring studies reviewed next in this paper tend to smooth out some of this inherent variability through biweekly or monthly composite sampling and by averaging data from local sites in an attempt to derive representative annual rates.

Data on time and space variation in concentrations of particles in the ambient air provide direct confirmation of the relative scales of variation that will ultimately affect the annual P deposition rates summarized in Tables 2 and 3. Graham and Duce[26] reported that P concentrations in the atmosphere over cities range from 30 to 1450 ng P $m^{-3}$, with an average of about 150 ng P $m^{-3}$, while values in coastal environments tend to be lower, in the range of 10 to 20 ng P $m^{-3}$. Low P levels, sometimes below 1 ng P $m^{-3}$, were also reported by these authors for remote marine and continental areas. Brook et al.[67] reported median concentrations for Canadian cities from 10 to 36 ng P $m^{-3}$ for particles less than 10 µm; concentrations for total suspended particles can be expected to be about twice these amounts. Prospero et al.[79], mentioned above, documented order of magnitude variations associated with seasons and climatic cycles in concentration data from Barbados. In essence, concentration data tend to follow expectations based on particle sources and transport: higher values in developed areas and low concentrations in undisturbed, remote environments.

Capturing the variability of P deposition associated with landscape patchiness remains a challenge. Plant canopies in aquatic or terrestrial systems can be expected to increase particle capture, but this effect might be less significant for giant particles than for pollutants conveyed by fine aerosols. In any case, scaling factors to translate data from surrogate surface collections into rates for various cover types in a regional landscape mosaic are not currently available. For dry P deposition driven by coarse and giant particles (Table 1), variability generated by rare, windy atmospheric events must be quantified. Annual rates could be more affected by episodic inputs than by more common deposition rates during fair weather.

**TABLE 2**
**A Summary of Annual Rates of Bulk P Deposition from the Atmosphere***

| P Deposition Rate (mg P m$^{-2}$ year$^{-1}$) Mean, Range, Sampling Period | N Deposition Rate (mg N m$^{-2}$ year$^{-1}$) | Sampling Location and Cover Type | Notes on Sampling Design and Techniques | Sources |
|---|---|---|---|---|
| 12.4<br>8.8–17.6<br>6 years | 795 | Shagawa Lake, Minnesota; forested, small-town setting | Bulk precipitation samples at a single station in the city of Ely, MN; bulk concentrations X monthly precipitation on lake | [129] |
| 14.<br>6–22<br>5 years | 490 | Northern inland, various forested sites | Bulk deposition (assumed), eight stations, 5-year period, 1971–1976 | [99] (Table 1) |
| 15.<br>12–18<br>15 years | 1020 | Slapy Reservoir, Czech Republic; forested | Bulk collector, one station, sampled after precipitation events (site I), 4 m above maximum water level | [95] |
| 15.6<br>6.9–22.8<br>6 years | None | Chamela tropical dry forest, Mexico | Bulk collectors with funnels, six stations in cleared areas, glass wool in funnels, storm event sampling | [130] |
| 19.<br>None<br>1 year | None | Coastal shrub habitat, South Africa | Five replicate collectors with 1 µm screens, 1.5 m above soil surface, weekly sampling interval; local soils very low in phosphorus | [131] |
| 20.3<br>17.8–23.7<br>3 years | 424 | Narrow Lake, Alberta, Canada; forested | Three to five bulk collectors, sampled after events and dry periods, funnels on collectors | [94] |
| 23.8<br>11.1–74.9<br>16 years | 1103 | Lakes in Central Ontario, Canada; forested | Various sites, 1976–1992, bulk collections with window screens, sampling median 8 days | [132] |
| 24.<br>12–31<br>13 years | 1380 | Rimov Reservoir, Czech Republic; farmland, village | Bulk collector, single station, sampled after precipitation events (site II), 14 m above maximum water level | [95] |
| 27.7<br>16.9–55.8<br>(1.5 years) | None | Lake Michigan area; urban settings | Data from ref. Fig. 2; 15 sites monthly for 18 months, sampled bulk collections | [93] |
| 32.5<br>24–53<br>4 years | 625 | Rawson Lake, Ontario, Canada; forested | Single station on island in lake, 4 years of data, bulk collections with 1.5-mm screening | [2] |

**TABLE 2 (Continued)**
**A Summary of Annual Rates of Bulk P Deposition from the Atmosphere***

| P Deposition Rate (mg P $m^{-2}$ $year^{-1}$) Mean, Range, Sampling Period | N Deposition Rate (mg N $m^{-2}$ $year^{-1}$) | Sampling Location and Cover Type | Notes on Sampling Design and Techniques | Sources |
|---|---|---|---|---|
| 35.8<br>No range<br>1 year | 587 | Piburger See, Austria; forested | Five stations on lake for large material + ten for fine; biweekly, bulk samples, large samplers + 1-mm screen, small w/o screens | [74] |
| 40.1<br>38.3–42.0<br>2.5 years | None | Mediterranean Sea, Corsica Island; marine | Single station on the coast, 10-m tower, 2.5 years of data, bulk collections, sampling period 15 days | [97] |
| 40.7<br>11.3–76.6<br>5 years | None | Various land uses, Southern Florida | 13 stations with Aerochem® wet/dry collectors, extensive data screening and uncertainty analysis; Std. Dev. = 33 mg P; range reflects site means for 5 years | [21] |
| 41.0<br>27.4–70.6<br>12 years | 1176 | Chesapeake Bay; rural, forested, wetland | Single-site event sampling since 1978 for TN and 1980 for TP, bulk collections, 13-m tower (Baltimore/Washington D.C. airshed) | [35] |
| 50<br>17–96<br>1 year | 750 | Florida, coastal, rural, agricultural and urban | 24 sites within the state, sampled biweekly, bulk collections using buckets for 12 months in 1978/79 | [23] |
| 59<br>24–96<br>1 year | 910 | Florida, urban, maritime, rural and agricultural | Sites at 4 locations in Florida, sampled biweekly, wet/dry buckets, Aerochem® wet/dry collectors, 12 months 1978/79 | [23] |
| 62.0<br>16–118<br>5 years | None | Northern Everglades marsh sites | Four marsh sites with Aerochem® wet/dry collectors, weekly samples, extensive contamination screening; range applies to site averages | [42] |
| 93.3<br>52–176<br>1 year | 859 | Tampa Bay Watershed; misc. land uses | Seven stations, sampled biweekly, bulk collections using funnel samplers; value is mean of seven stations | [24] |

* Deposition values are from published data using bulk or wet/dry collectors for periods from about 1 to over 20 years. These examples are based on data from either multiple years at a site or multiple collectors for at least 1 year to illustrate typical rates for various locations on an annual basis.

**TABLE 3**
**A Compilation of Average Rates of Atmospheric P Deposition from Published Literature Summaries on Bulk Atmospheric Deposition***

| P Deposition Rate (mg P $m^{-2}$ $year^{-1}$) Mean, Range | Sampling Location and Cover Type | Notes on Sampling Design and Techniques; Misc. Comments | References (Figures or Tables) |
|---|---|---|---|
| 23.8<br>3.6–60 | Mean for 17 forests in U.S. and Western Europe | Various annual mass balance studies of key elements, primarily bulk collections | [28] (Table 24) |
| 26.<br>5–60 + | 45 literature values for P inputs worldwide | Weighted mean based on ref. Fig. 4; some values above 60 not included in figure; based on summary tables or cases published in the literature; 1-year value of 22 from Gibson et al. not included | [29] (Fig. 4) |
| 30.<br>7–150 | North America, continental average | 25 authors summarized in ref. Table 3; average given in ref. Table 6, various approaches to measurements, 1960–1976 | [26] (Table 3) |
| 33.8<br>8.9–102 | Great Lakes, regional mean | Nine annual means, six studies, bulk deposition samples, monthly data | [93] |
| 35.9<br>12.4–93.3 | Typical value for aquatic ecosystems | Average of 17 values, each representing multiple annual measurements of bulk deposition for a site or region; data in Table 2, this study | This study (Table 2) |
| 50.<br>20–200 | Western Europe, regional average | Average from 16 authors reviewed in ref. Table 4, average from ref. Table 6; bulk deposition data 1951–1976 | [26] (Table 6) |
| 65.7<br>7–170 | P input to ecosystems worldwide | Mean deposition rate for 20 sites compiled after scrutinizing for soundness; most from North America and Western Europe; bulk measurements with funnels | [27] (Table 5) |

* Each mean value in this table represents multiple years of data summarized by the authors and can be considered as a representative regional or continental average for deposition rates within the last 3 decades.

## PUBLISHED RATES FOR P DEPOSITION FROM THE ATMOSPHERE

The second objective of this study was to summarize P deposition rates from the scientific literature in light of the conceptual framework provided above and to assess these estimates for applicability to environmental management and to South Florida specifically. There are hundreds of annual values for atmospheric P deposition published in the literature. This investigation seeks to build on earlier literature reviews[26,27,28,29,93,99] and provide a synoptic view of annual rates to bracket long-term values that can be expected in aquatic ecosystems surrounded by various land uses.

The vast majority of published rates of P deposition are gross estimates of deposition from *bulk* measurements in which the sampler, often a funnel, is left exposed to both wet and dry deposition for extended periods, usually 1 to 2 weeks. Atmospheric scientists are often critical of deposition estimates from bulk precipitation collections, citing several fundamental methodological limitations. Bulk collectors and the common Aerochem Metrics® wet/dry collectors are not designed aerodynamically, so that smaller particles will not be sampled efficiently, and captured particles may be resuspended from dry buckets. All surrogate surface sampling can be influenced to varying degrees by local contamination. This constraint, plus problems associated with the geometry and aerodynamics of the collector, must be recognized as these data are considered. While highlighting these considerations, Hicks et al.[12] note that bulk collectors or other artificial surfaces become more efficient as the particle size increases into coarse and giant particles controlled by sedimentation from the atmosphere. While the drawbacks of bulk collections are undeniable[100], the lack of practical alternatives for long-term sampling of coarse and giant particles encourages many investigators to continue publishing bulk deposition data or values from the common wet/dry bucket approach. The evidence compiled in this study will establish that many published bulk rates for P deposition are in general agreement with data derived from other independent approaches to estimating deposition, and tend to be internally consistent in range and average magnitude for various land uses. Until estimates of P deposition are available in a variety of ecosystems using other more rigorous methods, bulk or wet/dry deposition data provide the only estimates of long-term atmospheric inputs for most management purposes.

Annual mean rates of P deposition for specific locations and using bulk and Aerochem Metrics® wet/dry measurements range from 12.4 to 93.3 mg P $m^{-2}$ $year^{-1}$ (Table 2). Regional mean P deposition rates reflected in earlier literature summaries on the subject range from 23.8 to 65.7 mg P $m^{-2}$ $year^{-1}$. Although the complete range of published annual values in the cited references for both tables is from 3.6 to 200 mg P $m^{-2}$ $year^{-1}$, most published bulk P deposition rates fall between 20 and 80 mg P $m^{-2}$ $year^{-1}$. An earlier summary of 45 published values by Gibson et al.[29] yielded a smaller range in annual rates (5 to 60 mg P $m^{-2}$ $year^{-1}$). His summary had a mean of 26 mg P $m^{-2}$ $year^{-1}$, which is very close to the mean value of 34.8 mg P $m^{-2}$ $year^{-1}$ in Table 2 of this study. Newman[27] scrutinized published bulk deposition values from funnel collectors where technically sound methods were documented. Compared with local values in this study (Table 2), Newman's data resulted in a higher mean of 65.7 mg P $m^{-2}$ $year^{-1}$, with a nearly identical range of 7 to 170 mg P $m^{-2}$ $year^{-1}$. Only two sources of data were used in both Newman[27] and this study: Graham and Duce[26] and Schindler et al.[2].

In marine environments, deposition tends to occur at relatively low rates in the offshore environment despite the P-enriched surface microlayer that can be a source of atmospheric P[10]. For example, Graham and Duce[26] reported a value of 7 mg P $m^{-2}$ $year^{-1}$ from coastal Rhode Island, and in Florida, Hendry et al.[23] (1981) reported a low deposition rate of about 17 mg P $m^{-2}$ $year^{-1}$ for a maritime station in the Florida Keys. Guerzoni et al.[9] concluded that P deposition to the Mediterranean Sea ranges from about 20 to 40 mg P $m^{-2}$ $year^{-1}$, with higher values near the shore. For the southeast Mediterranean Sea, Herut et al.[101] estimated the dry deposition rate of inorganic P at 40 mg P $m^{-2}$ $year^{-1}$.

Inland sites associated with northern forests of the Midwestern U.S. or Canada, and one site from a tropical dry forest in Mexico, tend to have bulk deposition rates of less than about 40 mg P $m^{-2}$ $year^{-1}$. Rates in the middle of the range (30 to 50 mg P $m^{-2}$ $year^{-1}$) represent a variety of aquatic systems surrounded by mixed land uses (Tables 2 and 3). Values of 50 or more tend to be associated with productive ecosystem sites or agricultural and urban land uses. The mean total N:P mass ratio in annual values of Table 2 is 25, and the N:P ratios tend to decrease as P deposition rates increase. Thus, P limitation of primary productivity will tend to be maintained or enhanced by atmospheric nutrient inputs in less-disturbed forested landscapes. Jassby et al.[5] concluded that the N:P

relationship in atmospheric deposition to Lake Tahoe was consistent with the observed shift from N to P limitation in this oligotrophic, subalpine lake.

Together, the information on mean rates and ranges in Tables 2 and 3 provides gross estimates of P deposition useful for assessing the relative importance of atmospheric inputs into ecosystems. However, it is vital to recognize that the values compiled in Tables 2 through 5 are published arithmetic means and represent upper bound deposition rates for the various locations being studied. The

**TABLE 4**
**A Summary of Rates of Atmospheric P Deposition from the Scientific Literature for Remote Areas of the Globe***

| P Deposition Rate (mg P $m^{-2}$ $year^{-1}$) | Location, Sources and Other Notes | Notes on Sampling Design and Techniques; Misc. Comments | References (Figures or Tables) |
|---|---|---|---|
| 0.9 | P input from dust transported from Asia to the Hawaiian Islands | Based on the application of geochemical tracers to separate atmospheric dust from Hawaiian basalt soils; dust is transported over 6,000 km from Asia | [105] |
| 1.1–4.7 | Saharan dust delivered to the Amazon Basin | Trajectory analysis of air masses used with concentration data and percent mass of Saharan dust to estimate a deposition rate for P into the Amazon Basin, South America | [133] (Table 4) |
| 3.2 | P deposition to N.E. Spain in "red rains" from North Africa | Based on data from Catalonia, Spain collected over 11 years; isentropic back trajectories used to identify sources and P averaged 0.063% of North African dust | [80] |
| 5–6 | Deposition rate for remote areas in Northern Hemisphere | Based on data from U.S., Canada, Finland, Russia, Sweden, and other sites | [134] (Tables 3 and 4) |
| 13 | P accumulation rate for raised bogs in Southern Sweden | Based on recent rates in the upper 4 cm of sediment cores; rate four- to sixfold higher than that for preindustrial period (2 to 3 mg P $m^{-2}$ $year^{-1}$) | [134] (Table 5) |
| 21.5 | Preindustrial P accumulation rate at site TS7 in the Taylor Slough drainage of the Southern Florida Everglades | Based on mean accumulation rate for sections of a sediment core below 6 cm, dated at 1892; one high value at 12.5 cm excluded. Other nearby sites had pre-1900 mean accumulation rates from 17 to 78 P $m^{-2}$ $year^{-1}$; William Orem (personal communication) recommended TS7 as an undisturbed site supported almost solely by rainfall. Upper two sections of core, post-industrial period, averaged 26.1 mg P $m^{-2}$ $year^{-1}$ | [135] (data from Tables 10 and 11) |

* Each value in this table represents an annual estimate using a variety of methods as described. These values can be viewed as minimal, long-distance, long-term P deposition rates from the atmosphere.

**TABLE 5**
**A Summary of P Deposition Rates Estimated using Methods Other than Bulk Deposition or Wet/Dry Bucket Collections***

| P Deposition Rate (mg P $m^{-2}$ $year^{-1}$) | Location, Sources and Other Notes | Notes on Sampling Design and Techniques; Misc. Comments | References (Figures or Tables) |
|---|---|---|---|
| 32.5 (a)<br>15.6 (b) | North Yorkshire, England, rural setting | Rates from 17 monthly dust collections; mean data from duplicate wet inverted Frisbees; dust mass assumed to contain 0.001P; stations near Barlow (a) and Smeaton (b) | [117] (Tables 1 (a) and 2 (b)) |
| 135. | Urban site, Chicago, IL | Based on particle mass and size data from a flat plate with Mylar strips and concentration/size data from Wide-Range Aerosol Classifier; multistep calculation method used with 21 days of summer data; rate assumes particles contain $p$ = 0.001mass; dry deposition only | [56] (Table 3) |
| 94.6 | Urban site, Chicago, IL | Based on 17 summer samples, both day and night; mass flux by size from a flat deposition plate with Mylar strips and concentration data from a Noll Rotary Impactor; rate assumes $p$ = 0.001 mass; with upper and lower surfaces included, deposition rate would be 126.1 mg P $m^{-2}$ $year^{-1}$; dry deposition only | [32] |
| 15. | Ninety Mile Beach, coast of North Island, New Zealand | Based on 3-month study in 1983: dry deposition to a 10-cm Petri dish on 11-m tower from five, 50-h samples; wet deposition via funnel in seven events; dry data biased low due to smooth surface and summer collections only with onshore winds | [64] |
| 47 (a)<br>14.6 (b)<br>9.9 (c) | a. Urban, Chicago<br>b. Rural, South Haven<br>c. Aquatic, Lake Michigan | Based on three sampling periods in July and August, 1991 in Illinois with Noll Rotary Impactor for concentration and flat plate sampler for size-specific flux. Multistep method used to calculate mass flux; converted at $p$ = 0.001mass; rates conservative due to summer sampling and no wet deposition data | [55] |
| 5.9 | Aquatic site, Southern Lake Michigan | Data from 3 to 5 days per month from May through September 1977; high-volume sampler and three-stage cascade impactor, plus meteorological data; mean deposition velocity and concentration used for rates; rate biased low due to the exclusion of giant particles, low deposition velocities, summer data only, and no wet deposition data; probable rate = >15 mg P $m^{-2}$ $year^{-1}$ | [136] (Table 6) |

* Most estimates are derived from variations of the inferential technique using air concentration data and deposition velocities. Note that several of the rates for P are conversions from total particle mass using a value of P mass = 0.001 total mass, a conservative average for crustal materials.

use of geometric means or medians in the original data would reduce these values, particularly in those data sets subject to high variability. Considering this caveat, and using South Florida as a case study, we should expect values less than 20 mg P $m^{-2}$ $year^{-1}$ for sparsely populated areas near the Florida coast receiving mostly onshore winds. Inland rates will vary with land use and distance from sources. High P inputs (60 to 100 + mg P $m^{-2}$ $year^{-1}$) can be anticipated near urban or agricultural areas, dropping off as one moves into open-water areas of the Everglades; Hendry et al.[23] reported the high values for urban (48 mg P $m^{-2}$ $year^{-1}$) and agricultural (66 mg P $m^{-2}$ $year^{-1}$) areas of Florida.

A spatially integrated rate for the remnant Everglades marshes must consider urban inputs from the east, agricultural sources to the north and east, and maritime areas to the south and west (Fig. 1). It is reasonable to expect low deposition rates over large areas of Everglades National Park and Water Conservation Area 3, distant from local sources, and higher rates in the northern parts of the remnant system, including Water Conservation Areas 1 and 2. With these facts in mind, a spatially integrated, long-term average input of atmospheric P to Everglades marshes, known collectively as the Everglades Protection Area, should fall between 30 and 40 mg P $m^{-2}$ $year^{-1}$, based on published estimates of deposition in the literature. McDowell et al.[102] also arrived at this range based on their analysis of P deposition into South Florida. The upper bound of this range is close to the 40.9 mg P $m^{-2}$ $year^{-1}$ 13-station mean of Ahn and James[21]. The values developed by Walker[42] in the WCA-1 marsh (Fig. 1) bracket rates expected as background (ca. 20 mg P $m^{-2}$ $year^{-1}$) and as levels associated with high anthropogenic inputs (ca. 100 + mg P $m^{-2}$ $year^{-1}$). The earlier 37 mg P $m^{-2}$ $year^{-1}$ estimate used by Walker[103] for the Northern Everglades appears to be in general agreement with values summarized in this study. Finally, a value of 30 mg P $m^{-2}$ $year^{-1}$ was used by Fitz and Sklar[104] in a spatially explicit ecosystem model of Water Conservation Area 2A in the Northern Everglades. Their model was found to provide good agreement with observed data, including data on P dynamics in WCA-2A.

P deposition rates for remote areas of the globe illustrate that there is no place in the world where one would expect the rate of P deposition from the atmosphere to be zero (Table 4). There is evidence that areas thousands of kilometers from Asian or African sources of dust still receive from about 1 to 5 mg P $m^{-2}$ $year^{-1}$ from atmospheric transport. Although these estimates seem small, they can be significant over long periods in oligotrophic tropical settings[105]. The two largest background values are based on long-term sediment P accumulation rates from the Hawaiian Islands and the Amazon Basin, receiving P loading almost entirely from the atmosphere by long-distance transport (Table 4). The rates at the bottom of Table 4 derived from sediment P accumulation rates are less than about 22 mg P $m^{-2}$ $year^{-1}$ and overlap with the range of values seen in remote marine and forested settings over the last few decades (Tables 2 and 3).

Recent sediment P accumulation values for the unenriched sections of Florida Everglades marshes in Craft and Richardson[106] and Reddy et al.[107] are higher at about 70 and 150 mg P $m^{-2}$ $year^{-1}$, respectively. These rates were not included as background values because they have been influenced to some uncertain, but significant, extent by water management, associated inputs of nutrient-rich stormwater and sediment P cycling. Walker[103] used these recent P accumulation data to derive a relationship between P accretion and distance from surface inflows in WCA-2 (Fig. 1). As P concentrations fall to near-background levels over 10 km from inflows, Walker's model predicts P accretion rates of about 75 mg P $m^{-2}$ $year^{-1}$. The proportion of nonatmospheric loading in this rate is again uncertain, but atmospheric P deposition clearly must be substantially less than this value over vast areas of the Everglades far from the influence of surface inflows.

The bulk deposition values (Tables 2 and 3) are also consistent with rates derived from techniques using concentration data and size-specific deposition velocities, inverted Frisbee collectors or Petri dish samples combined with funnel sampling of rain (Table 5). Data from urban Chicago sites range from 47 to 135 mg P $m^{-2}$ $year^{-1}$, rural sites range from 14.6 to 32.5 and aquatic sites range from 5.9 to 15 (both rates are biased low)(Table 5). Most of these data for concentrations and settling velocities to flat surfaces are for short sampling periods, and extrapolation to annual values is tenuous. Overall, the values of Tables 4 and 5 support the conclusion that inherent uncertainties in estimates from bulk or Aerochem Metrics® wet/dry collections do not preclude the use of this approach for setting crude bounds on long-term atmospheric P deposition rates expected for various land uses.

## FUTURE MEASUREMENTS OF ATMOSPHERIC P DEPOSITION

Are the values compiled in this study useful as rough estimates for decision making in ecosystem management or research? To address this question, each manager will have to assess the potential importance of atmospheric inputs to decide the technical effort to expend in reducing the uncertainty of P inputs from the atmosphere. Regarding South Florida, the majority of P loading to the pristine portions of the Everglades ecosystem comes directly from the atmosphere, and the remnant system is, as a whole, very oligotrophic. In fact, there is strong evidence that a P water quality criterion for the ecosystem of about 10 μg $Pl^{-1}$ is scientifically defensible[44], and major changes in P deposition could alter water column P concentrations in this system. Based purely on the important role of P in this ecosystem, more accurate and precise information would be desirable. However, this need for better information must be tempered by practicalities. From a resource-management perspective, most atmospheric nonpoint source inputs of P-bearing particles are not readily abatable, at least on a regional level. If the rates compiled to date are found to be unrepresentative and overly conservative for localized sources from industry, agriculture, or urban areas through mass balance studies or water quality monitoring, then further quantification might be considered for the Everglades or any other managed ecosystem. The second practical consideration is the challenge of improving upon existing bulk or wet/dry data with methods suitable for long-term quantification; this aspect is considered next.

When additional measurements are warranted for a particular ecosystem, how should they be done? Published bulk precipitation or Aerochem Metrics® wet/dry measurements provide rough rate estimates, but the drawbacks of this approach, particularly contamination and inherent characteristics of bucket collectors, suggest that additional data will be of very little value in improving the predictability of P deposition rates. Proven alternatives for long-term monitoring of coarse and giant particles are limited, however, because recent methodological advances in long atmospheric environmental measurements have focused on acid precipitation and other pollutants with gaseous forms or in fine aerosols[12,34]. There are promising methods for quantifying coarse and giant particles, but none have been used on a multiyear basis or perfected for application in remote locations.

The analysis that follows attempts to guide the environmental manager through available methods to arrive at recommendations on how to improve estimates of P deposition rates from the atmosphere, particularly for the dry component. The evaluation is organized around the recommendations of Hicks et al.[12], Erisman et al.[86], Lovett[34], and McDowell et al.[102] These authors emphasize that: wet and dry deposition should be monitored separately; dry deposition conveys most atmospheric loading; and estimates of P deposition will be most improved by using several complementary techniques simultaneously.

### Wet Deposition of Phosphorus

Automatic wet/dry deposition collectors are recommended for wet deposition sampling[86]. The Aerochem Metrics®, Inc. samplers consist of a wet and dry bucket, each 29.4 cm in diameter. The wet bucket is uncovered only during periods of rainfall, while the dry bucket is left open all the time, except for periods of active precipitation. For wet deposition, wet/dry collectors appear to provide representative samples without the aerodynamics drawbacks associated with dry deposition. The National Atmospheric Deposition Program (NADP) recommends the Aerochem Metrics®

301 Collector for its nationwide network of wet deposition sites, and the device has been used widely in other studies of wet deposition[5,21,23,25,35,42]. The wet bucket collects rainfall in amounts very close to those attained by independent rain gauges, and there are no cases of systematic bias in these samplers for rainfall water quality and quantity reported in the deposition literature.

Challenges remain, however, in using Aerochem Metrics®, Inc., collectors for wet deposition. In active wildlife areas, there will always be some contaminated samples from biological debris that could dissolve in the collector and go undetected in field processing. Still, rigorous quality assurance procedures can help reduce and identify unrepresentative samples. Mounting collectors above the ground could help to avoid large local particles and minimize biofouling: McDowell et al.[102] recommend sampling at a 10-m height. A recent study by Pollman et al.[108] was very successful at reducing contamination and local particle inference by using a series of sampling improvements and replicated containers within the Aerochem Metrics®, Inc., collector mounted on 10-m towers in Florida. This study shows the value of careful field and laboratory techniques in deposition studies. Even with better quality controls, procedures are still needed to deal with aberrant wet samples; Ahn and James[21], Pollman et al.[108], and Walker[42] provide examples of statistical approaches to analyzing deposition data.

## Dry Deposition of P-Bearing Particles

Common methods for estimating the dry deposition of particles include three general approaches to measurement: micrometeorological methods, surface accumulation methods, and inferential techniques. Reviews by Hicks[91], Nicholson[109], Erisman et al.[86], Hicks et al.[12], and Lovett[34] provide detailed comparisons of various methods for assessing gaseous, aerosol, and particulate deposition. The following summary is intended only to highlight key facts on each technique, with emphasis on particulate measurements applicable to particles conveying P, and with consideration of the potential for routine quantification for future environmental studies.

### *Micrometeorological Methods*

Micrometeorological methods have been successful in quantifying the submicron aerosol component of long-term ecosystem inputs for such pollutants as ozone, sulfate, methane, and ammonia[30,34]. These methods can be applied *in situ*, provide continuous information, and do not disturb the sampling site. Micrometeorological methods use several approaches to estimate flux rates from air concentrations and meteorological data[30,86,91]. Baldocchi et al.[30] and Nicholson[109] give a summary of the fundamental theory for these approaches.

Unfortunately, these techniques as currently developed are not suitable for particles, even fine particles, above 1 µm in size[86,91]. Sampling gradients and directional fluxes of larger, rapidly settling particles is extremely difficult or impossible[109]. Documenting no more than a few percent of dry P deposition with current micrometeorological methods from very fine (submicron) particles cannot justify the investment needed for measurements. This conclusion might not hold true for cases in which the deposition of gaseous P must be measured. Micrometeorological methods are scientifically appealing because they attempt to directly quantify atmospheric exchange processes, allowing estimates at the landscape level. They might become more useful if advances in instrumentation make them more widely applicable to P-bearing supermicron particles.

### *Surface Accumulation Methods*

Surface accumulation methods provide accumulation rates from mass changes directly on surfaces, natural or surrogate[86,91,109]. These methods hold the greatest promise for long-term monitoring of P deposition. Surface-accumulation studies have proven more useful for coarse (2.5 to 10 μm) and giant particles (> 10 μm), whose deposition is governed by gravitational forces[55,56,65]. Most sizes of atmospheric particles accumulate on downward- and upward-facing surfaces due to the concurrent effects of inertia and gravity[32]. As a result of this complexity, interpreting data for a particular surface area and collector shape is not straightforward. Combinations of scaled surrogate surface accumulation measurements might eventually be appropriate for environmental monitoring; calibration to estimate P deposition onto complex natural surfaces is needed.

### *Net Throughfall*

Differences between total bulk deposition in an open area and under a canopy estimate net dry deposition to a natural plant community in what is termed “net throughfall”. Throughfall measurements reflect the sum of wet, dry, cloud, and dew deposition onto natural plant communities[34,86]. This technique has been used successfully to estimate net deposition fluxes to forest canopies[76,90,110,111]. Vegetative processes (foliar leaching or uptake) can affect data interpretation, and P enrichment of precipitation moving through tree canopies has been documented[112,113,114]. Such canopy processes are likely to confound throughfall P measurements, and this source of variation, plus the physical difficulty with *in situ* canopy and bulk measurements in wetland settings, make net throughfall an unlikely candidate for P deposition monitoring. If throughfall studies are found to be useful for P, their primary purpose will be to calibrate other approaches to long-term data collection for particular canopy types.

### *Frisbee-Shaped Collectors*

To overcome aerodynamic interference with depositing particles, inverted Frisbee-shaped airfoils have been used for particle flux measurements[115,116]. Net mass accumulation to flat surfaces inside the sampler is quantified over specified periods of time, and these Frisbee-shaped collectors have been used effectively in combination with other sampling devices in estimating dry deposition of a diverse suite of parameters[33,57]. These aerodynamically shaped devices offer advantages for deposition monitoring, and they have been used in the U.K. for dust monitoring[117]. Resulting data give reasonable estimates of deposition rates, as long as coated surfaces are used; these estimates are viewed as minimal rates to complex natural surfaces. Wind losses, changes in surface stickiness, and contamination events are potential drawbacks.

### *Flat Plate Sampler*

Another device that has good aerodynamic qualities is the flat plate sampler with a wind vane developed by Noll et al.[32] This plate sampler provides reproducible flux estimates to a flat, greased surface and is well suited to both chemical and numerical studies of particles[63]. Using coatings in a dry collector increases capture efficiency and might provide better estimates for aquatic systems. In productive aquatic settings, however, either of these devices may be subject to contamination by insects and birds. Field trials are needed to compare and calibrate methods for use in routine monitoring of P deposition, but the cited studies suggest that these collectors offer promise for routine deposition monitoring.

### *Integrated Throughfall, Surrogate Surface and Bulk Sampling*

Long-term monitoring of chemical deposition to forests has been accomplished using artificial surfaces designed to simulate a canopy (Teflon string), with calibration by throughfall collections and bulk deposition[118]. It uses the method developed by Ferm and Hultberg[119] for forest deposition monitoring in Sweden. The dry deposition of an ion is calculated by multiplying the ratio of sodium deposited on the string device to that in throughfall by the amount of the ion deposited on the string collector. This technique is attractive in that it assesses inputs at the canopy level without using electricity. With multiple sites, it is suitable for long-term data collection at the regional level to assess critical loads of pollutants. Variations of this integrated approach using various other artificial surfaces have been applied to forest deposition[118].

This integrated method is cost effective once the artificial surface has been shown to collect a target chemical species; it has provided results for N and sulfur deposition comparable to other estimates. Determining the utility of the method for coarse and giant particles would require additional research. Contamination issues for bulk measurements and throughfall sampling remain significant uncertainties, particularly in productive aquatic environments. Covering the surrogate surface collector might help with sampler contamination, but its effect on particle capture would need to be studied. Even with these challenges, the concept of using a convenient surrogate collector calibrated by throughfall studies has potential for large-scale, long-term monitoring of P deposition.

### *Bulk Funnels, Wet/Dry Buckets and Other Artificial Surfaces*

Bulk and wet/dry Aerochem Metrics®, Inc. collectors are the most common monitoring devices for atmospheric deposition of P; many references are given in Tables 2 and 3. Reviews of deposition methods provide examples of other diverse surfaces that have been used to estimate bulk atmospheric inputs[34,86,91]. As particle size increases, these collectors give better estimates of flux[86,91], although bulk measurements tend to underestimate inputs of ions conveyed by vapor or fine particles into forest canopies[110]. Davidson et al.[65] and Lovett[34] provide a guide to the literature on attempts to use many other artificial surfaces in deposition studies, and they note that artificial surfaces might be the best available method to assess deposition rates of large particles.

Some inherent deficiencies exist in surrogate surface collectors, such as the common Aerochem Metrics®, Inc. dustfall buckets for collecting quantitative samples of dry deposition. Particle resuspension, bounce-off, and contamination are always possible for artificial surfaces. Birds are a particular challenge to deposition measurements in marshes[39,40,42]. The geometry of dustfall buckets is also a problem, according to Hicks[91] and Davidson et al.[65], because buckets have a high surface:bottom area ratio of 4.8, making dry deposition data difficult to interpret as deposition per unit surface area of a canopy or the ground. In addition, bucket geometry distorts airflow at the bucket rim and causes particles to change their trajectories and escape capture by the bucket[120]. As pointed out by Harrison and Perry[59], even larger particles tend to fall at very small angles and can easily be diverted away from surfaces by airflow characteristics of the bucket rim. Further, the chemical and physical nature of the surface can also be a factor in observed mass accumulations[121,122].

Together, these considerations make the interpretation of dry P deposition data from dustfall buckets and bulk collectors very difficult[100,102,109]. The evidence reviewed in this study suggests these uncertainties are not large enough to preclude using bulk deposition and data from wet/dry collectors to bracket grossly the deposition rates for P (Tables 2 and 3). For study areas with infrequent contamination where there is a need for a first estimate of P deposition, wet/dry collectors can used to provide a crude deposition rate. Adding water to the dry bucket can be considered to avoid resuspension and to get estimates closer to those experienced at the air-water interface[5,72].

### *Surface Accumulation Directly on Vegetation or Water Surfaces*

Mass accumulation over a period of several days on leaf surface has been combined with surrogate samplers to estimate coarse particle deposition to forest canopies[123]. Leaf surface provides a direct estimate of dry deposition but is subject to large uncertainties. Cloud/dew droplets can change rates. Foliar interactions with deposited P may be a substantial interference for estimates to a system with a plant canopy. Phosphorus accumulation on the surface of vegetation is probably not appropriate for routine monitoring of atmospheric deposition, but could be considered for validation and calibration purposes. Yi et al.[124] used a water surface sampler to measure dry deposition of sulfate. This surface sampler deserves further study, although it will be difficult to deploy in remote areas and will be subject to interference from strong winds and local contamination.

## *Inferential Techniques*

Inferential techniques estimate deposition rates from air concentration data used in combination with direct particle enumeration or models to estimate size-specific deposition velocities[91]. Inferential techniques vary greatly in their level of detail and resolution. They can provide reliable estimates for aerosols, gases, and fine particles for which air concentrations can be measured. Deposition velocities can be estimated by models driven by meteorological data[92,125,126]. Other variations on the inferential approach use direct particle enumeration and quantification of air concentrations for coarse and giant particles; these designs hold great promise as means of estimating dry deposition of particles conveying P into natural systems.

Many devices are available to collect fine and smaller coarse particles for compliance with ambient air standards[31], and air concentration data can be used to yield crude deposition estimates when combined with average velocities. The difficulty is getting air concentration and deposition velocity data for specific size classes, particularly coarse and giant particle classes. Various impactor samplers allow quantification of the concentration of particles from below 1 to about 100 μm[56,63]. Combining rates of particle accumulation from a standardized surface, such as a flat plate sampler[32], with air concentrations from impactor sampling can yield good estimates of dry deposition for P, as has been done by Davidson et al.[66] and Lin et al.[56] for other components of atmospheric deposition.

### *Low Volume Filter Packs and the NOAA, DRY DEP 2 Model*

Air concentrations of submicron particles and gases derived from filter pack samples and meteorological parameters are widely used to model deposition rates[92,125]. Models have been calibrated extensively for gaseous deposition[125,126]; validation of modeled parameters remains a major uncertainty[34]. By design, filter packs do not quantify larger coarse and giant particles. Consequently, the P deposition rates estimated with filter pack data and deposition models by Yang et al.[96] and Hu et al.[127] were unrealistically low, representing less than 10% of likely deposition rates. Overall, the approach is not useful for monitoring total P inputs unless larger particles can be sampled and modeled more effectively.

### *Rotary Impactors and Flat Plates*

Large volumes of air are sampled by a multistage impactor, and change in mass of each stage yields size-specific concentrations. The device is used in combination with flux measurement to flat plates to derive depositional velocities for particle size classes[32,63,128]. The Noll Rotary Impactor provides a distribution of mass in 10 size classes up to about 100 μm and associated concentrations. A flat plate is used to estimate size-specific mass flux to a coated surface. Supermicron particle concentrations are quantified and, together with flux rates from plate sampling, data from this approach should provide mass flux for size classes that can be added to estimate total atmospheric P deposition at a flat surface. Caffrey et al.[57] and Paode et al.[69] combine the Noll Rotary Impactor for air concentration data for coarse and giant particles and surrogate surface (Frisbee or flat plate) methods to estimate deposition velocities to derive dry deposition estimates. This approach has great potential for P deposition studies and routine monitoring once it is calibrated for various natural cover types.

## BEYOND BULK PRECIPITATION AND WET/DRY DEPOSITION ESTIMATES

Lovett[34] was referring to many airborne nutrients and pollutants when he concluded: "A major challenge for the future will be to reconcile differences among existing measurement methods for cloud and dry deposition, and to arrive at a suite of generally agreed-upon methods that are applicable to a broad range of sites and chemical substances." His words are true today for atmospheric P deposition.

A combination of the approaches discussed in the previous section is needed to improve measurements of dry deposition rates. The goal is typically to measure net P deposition to an ecosystem and there is currently no single technique that can give all the necessary information for determining and validating deposition rates for the size classes of particles conveying P. However, there is a scattered literature demonstrating great potential for integrative approaches that combine and compare data from several surface accumulation and inferential methods. Forest studies have had success combing artificial surfaces with throughfall collections and bulk deposition to yield canopy level deposition estimates[118,119]. Various combinations of methods supporting an inferential approach have also proven to be a rigorous means to estimate the dry deposition of particles[33,57,69].

The South Florida Water Management District recently contracted with a group of experts in atmospheric science to investigate the science of P deposition relevant to the Florida Everglades. An important end product of this effort was a study design that would reduce uncertainty in rates and begin the process of developing a new generation of monitoring methods. The final, integrated product of their research was a work plan for a 1-year field study to: (1) establish upper and lower bounds of dry P deposition; (2) inter-compare methods of estimation; and (3) calibrate fluxes to a vegetated Everglades canopy[45].

The research plan of Pollman et al.[45] uses three complementary data streams, with *state of the science* methods (Fig. 3). For one data stream, surrogate surface samplers (flat plates and Frisbee-shaped samplers) would provide flux estimates for various size classes of P to flat surfaces. These estimates are viewed as setting the lower bound for P deposition rates. For the second data stream, air concentrations for more than 12 different size classes of particles would be measured through impactor sampling (Micro-orifice Uniform Deposit Impactor and Noll Rotary Impactor). Data on wind speed/direction, temperature, solar radiation, and humidity would be combined with aerosol concentration data in modeling deposition velocities and estimating dry deposition flux from the second data stream. To validate and calibrate rates derived from fluxes to surrogate surfaces and particle concentrations in the air, the third component of the study would use throughfall measurements of P deposition to provide information on canopy scaling. Throughfall estimates in marsh plant communities would also be developed for a conservative tracer of P deposition, probably sodium. This element of the proposed study would effectively "ground-truth" P throughfall estimates and, ultimately, validate rates from surrogate surfaces, aerosol concentrations and inferential models. Wet P deposition would be determined with Aerochem Metrics®, Inc. wet/dry collectors using the methodological improvements of Pollman et al[108]. Together the sampling methods and sites would provide detailed data to constrain estimates of particle fluxes in relation to local sources and meteorology.

The Pollman et al.[45] study design dramatically illustrates that there is no simple means to improve upon extant P deposition data. The proposed study would require intense collaboration for 1 year of field sampling, moving well beyond most other studies involving multiple approaches to dry deposition that have been conducted for relatively brief periods of time[33,55,57]. The proposed approach is also noteworthy in addressing uncertainty in P deposition in a holistic and balanced manner using methods at the cutting edge of deposition-related science.

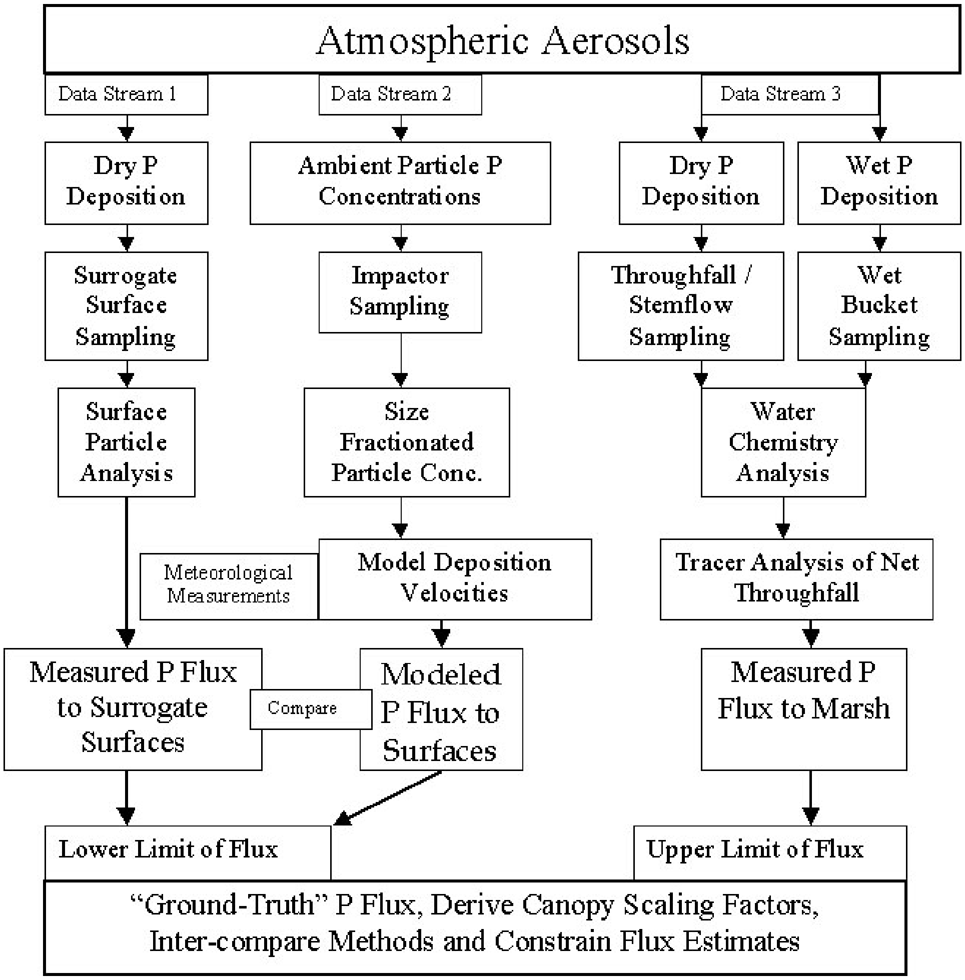

**FIGURE 3.** A diagrammatic representation of the experimental plan proposed by Pollman et al.[45] to improve estimates of P deposition. The plan features three data streams leading to three comparable sets of flux estimates. This figure is a major revision of Fig. 3 from Pollman et al.[45], and the author is solely responsible for its content.

## CONCLUSIONS

This review has attempted to bridge the disparate disciplines of environmental and atmospheric science to improve interdisciplinary understanding of P deposition from the atmosphere. Melding the information from a diverse literature in this study leads to the following conclusions and recommendations:

1. The vast majority of P in dry deposition is conveyed by coarse (2.5 to 10 μm) and giant (10 to 100 + μm) particles (Table 1). While fine particles (<2.5 μm) also contain P, their minute mass and slow settling rate makes them responsible for only a few percent of total dry deposition. The roles of phosphine and other forms of gaseous P remain highly uncertain. The coarse and giant components of deposition have been quantified far less than those in the fine size range, because other priority airborne pollutants tend to be conveyed in the fine fraction.
2. Processes operating across temporal and spatial scales at the land-air interface produce variation in P deposition from the atmosphere (Fig. 2). Dynamics in particle sources lead to variation in particle concentrations and P content and are strongly associated with land use. Important sources of P in airborne particles are fugitive soils, biological debris and pollen, ash, combustion products of fossil fuels, emissions from manufacturing, and aerosols from the surface of marine and freshwater ecosystems. Natural plant canopies capture particles and lead to variation in deposition rates across the landscape mosaic.

3. The only substantial information base on long-term P deposition rates is from bulk precipitation and wet/dry bucket collectors. These methods are subject to many constraints in both precision and accuracy. A compilation of annual published rates (Tables 2 and 3) reveals that many freshwater aquatic ecosystems receive a mean bulk deposition of about 20 and 80 mg P $m^{-2}$ $year^{-1}$. The overall range of published annual rates is from 3.6 to 200 mg P $m^{-2}$ $year^{-1}$. The median value for local rates is 30.1 mg P $m^{-2}$ $year^{-1}$ (Table 2), and for regional mean rates is 33.8 (Table 3).
4. P deposition occurs in measurable amounts even in remote locations of the globe (Table 4), and P can be transported thousands of kilometers from sources in Asia and North Africa. The magnitudes of values given in Tables 4 and 5 suggest that despite the inherent limitations in bulk or wet/dry sampling, these crude methods do estimate the upper bounds of annual P deposition from the atmosphere associated with various land uses.
5. The common wet/dry bucket sampling can be used to monitor wet deposition of P with stringent quality assurance measures in the field and laboratory. Contamination may produce some positive bias in estimates, but mounting samplers on towers and using other improvements will minimize this problem.
6. The comparative approach to dry P deposition measurement could constrain deposition estimates from the wide range found in this review and lead to new ways of conducting long-term, cost-effective monitoring. The likelihood of success is greatest using multiple data streams as proposed by Pollman et al.[45]. P flux in deposition is estimated directly using surrogate surfaces, and inferentially using aerosol concentrations from impactor sampling and models to estimate deposition velocities. Throughfall estimates of P flux can be validated using a conservative tracer and then used to scale rates to various natural canopies[45] (Fig. 3).
7. Contamination of samples in long-term monitoring will continue to cause some positive bias in estimates, even with field, laboratory, and statistical screening. As new methods are tested for P deposition sampling, reducing the frequency of contamination and increasing the likelihood of detecting unrepresentative samples must be primary considerations.
8. For South Florida's Everglades, information in this review leads to the conclusion that continued sampling with wet/dry bucket collectors will not result in improved precision or accuracy in wet or dry P deposition estimates compared to those derived from existing data[21,42]. The substantial investment in operating this monitoring network cannot be justified by uncertain information to be gained by future monitoring in these wetlands. New approaches to monitoring are needed if uncertainty in rate estimates for atmospheric P deposition is to be reduced (see #6).

Greater precision and accuracy in deposition rate estimates are fundamentally important to the certainty of decision making in eutrophication management worldwide and are most needed for environmental management of oligotrophic systems. In South Florida, as surface water nutrient loading to the Florida Everglades is reduced through implementation of the Everglades Forever Act and the recently authorized Comprehensive Everglades Restoration Plan, atmospheric loading will retake its historical place as a primary source of nutrients to most areas of South Florida's "river of grass". Hopefully, the conceptual model, literature review, and evaluation of methods presented above will contribute to an improved understanding of, and interest in, the transport and impact of airborne P in the Everglades and other aquatic ecosystems.

## ACKNOWLEDGEMENTS

This study could not have been completed without the support of the South Florida Water Management District, West Palm Beach, FL; I deeply appreciate this opportunity and challenge. I gratefully acknowledge the expert guidance provided by Drs. Tim Bechtel, Michael Chimney, Carl Fitz,

Thomas Fontaine, Susan Gray, Karl Havens, R. Thomas James, Dave Rudnick, and Alan Steinman on the earlier versions of the manuscript. Many thanks to Carrie L. Trutwin and Felicia Berger, who used their technical editing and grammatical expertise to fine-tune this document. The study benefited greatly from a synthesis of information on P deposition conducted for our agency by Drs. Curtis Pollman, Kenneth Noll, William Landing, and Steven Lindberg. Rachelle Grein and Christopher King collaborated to create Fig. 1. Christopher King provided graphics expertise for Fig. 2. The paper was improved by the thorough and constructive guidance of two expert referees.
